# Cytotoxicity of Zardaverine in Embryonal Rhabdomyosarcoma from a Costello Syndrome Patient

**DOI:** 10.3389/fonc.2017.00042

**Published:** 2017-04-03

**Authors:** Donna M. Cartledge, Katherine M. Robbins, Katherine M. Drake, Rachel Sternberg, Deborah L. Stabley, Karen W. Gripp, E. Anders Kolb, Katia Sol-Church, Andrew D. Napper

**Affiliations:** ^1^High-Throughput Screening and Drug Discovery Laboratory, Nemours Center for Childhood Cancer Research, Nemours Biomedical Research, Nemours/A.I. duPont Hospital for Children, Wilmington, DE, USA; ^2^Nemours Biomolecular Core Laboratory, Nemours Biomedical Research, Nemours/A.I. duPont Hospital for Children, Wilmington, DE, USA; ^3^Biological Sciences, University of Delaware, Newark, DE, USA; ^4^Division of Genetics, Nemours/A.I. duPont Hospital for Children, Wilmington, DE, USA; ^5^Nemours Center for Cancer and Blood Disorders, Nemours/A.I. duPont Hospital for Children, Wilmington, DE, USA

**Keywords:** sarcoma, Costello syndrome, high-throughput screening, phosphodiesterase, inhibitor, cytotoxicity, cell viability

## Abstract

Costello syndrome (CS) patients suffer from a very high 10% incidence of embryonal rhabdomyosarcoma (ERMS). As tools to discover targeted therapeutic leads, we used a CS patient-derived ERMS cell line (CS242 ERMS) harboring a homozygous p.G12A mutation in *HRAS*, and a control cell line derived from the same patient comprising non-malignant CS242 fibroblasts with a heterozygous p.G12A *HRAS* mutation. A library of 2,000 compounds with known pharmacological activities was screened for their effect on CS242 ERMS cell viability. Follow-up testing in a panel of cell lines revealed that various compounds originally developed for other indications were remarkably selective; notably, the phosphodiesterase (PDE) inhibitor zardaverine was at least 1,000-fold more potent in CS242 ERMS than in the patient-matched non-malignant CS242 fibroblasts, other ERMS, or normal fibroblasts. Chronic treatment with zardaverine led to the emergence of resistant cells, consistent with CS242 ERMS comprising a mixed population of cells. Many PDE inhibitors in addition to zardaverine were tested on CS242 ERMS, but almost all had no effect. Interestingly, zardaverine and analogs showed a similar cytotoxicity profile in CS242 ERMS and cervical carcinoma-derived HeLa cells, suggesting a mechanism of action common to both cell types that does not require the presence of an *HRAS* mutation (HeLa contains wild type *HRAS*). Two recent studies presented possible mechanistic explanations for the cytotoxicity of zardaverine in HeLa cells. One revealed that zardaverine inhibited a HeLa cell-based screen measuring glucocorticoid receptor (GR) activation; however, using engineered HeLa cells, we ruled out a specific effect of zardaverine on signaling through the GR. The second attributed zardaverine toxicity in HeLa cells to promotion of the interaction of phosphodiesterase 3A and the growth regulatory protein Schlafen 12. We speculate that this work may provide a possible mechanism for zardaverine action in CS242 ERMS, although we have not yet tested this hypothesis. In conclusion, we have identified zardaverine as a potent cytotoxic agent in a CS-derived ERMS cell line and in HeLa. Although we have ruled out some possibilities, the mechanism of action of zardaverine in CS242 ERMS remains to be determined.

## Introduction

Embryonal rhabdomyosarcoma (ERMS) is the most common pediatric soft tissue sarcoma, and its incidence is significantly increased in children with Costello syndrome (CS), a rare genetic disorder resulting from heterozygous germline mutations in the proto-oncogene *HRAS* located on 11p15.5 (1–5). ERMS associated with either p.G12S, p.G12C, or p.G12A mutations occurs with a very high lifetime incidence of 10% (6, 7). In the vast majority of CS-derived ERMS cases, patients carry a paternally inherited *HRAS* mutation and tumors display paternal uniparental disomy with loss of the maternally inherited chromosome 11 (8). The incomplete success to date of treating CS-derived ERMS patients highlights a need for better therapeutic options; out of 13 patients who received treatment, 3 died due to tumor progression or relapse (8).

Nemours has established a CS registry and tissue repository unparalleled in scope, allowing us to establish a CS patient-derived ERMS cell line harboring a homozygous p.G12A mutation in *HRAS* (CS242 ERMS), as well as a control cell line from the same patient comprising non-malignant CS242 fibroblasts with a heterozygous p.G12A mutation. Using these cell lines, we endeavored to discover compounds selectively toxic to ERMS harboring a homozygous *HRAS* mutation. Such compounds could lead to chemical probes to further understanding of the biological basis of malignancy in CS patients, and ultimately to therapeutic leads. We chose to screen using a phenotypic assay of cell viability rather than an assay explicitly targeted to HRAS signaling to provide insights into disease biology and therapeutic opportunities by uncovering active compounds with unanticipated molecular target(s) and mechanism of action.

We screened a library of FDA-approved drugs and other compounds that have undergone clinical testing to determine if any of these compounds could be “repurposed” to block ERMS harboring mutant *HRAS*. These compounds generally have good physicochemical properties, and many have been shown to modulate specific molecular targets, providing the basis for testable mechanism of action hypotheses. Zardaverine, previously developed as a phosphodiesterase (PDE) inhibitor (9, 10), displayed cell killing in CS-derived ERMS harboring homozygous mutant *HRAS*. Strikingly, zardaverine had no effect on the growth of any other cell lines tested with the exception of HeLa. Here, we describe characterization of the activity and selectivity of this compound, and initial studies to provide clues as to mechanism of action.

## Materials and Methods

### Materials

#### Expression Vectors and Transfection Reagents

GL4.36[luc2P/MMTV/Hygro], pGL4.54[luc2/TK], and pNL1.1.TK[Nluc/TK] were from Promega (Madison, WI, USA). Lipofectamine 3000 was from Life Technologies (Grand Island, NY, USA).

#### Cell Culture and Assay Development Supplies

Dulbecco’s modified Eagle’s medium (DMEM), phenol red-free DMEM, Roswell Park Memorial Institute (RPMI) 1640, phosphate-buffered saline (PBS) without calcium and magnesium, 0.25% trypsin in Hanks’ balanced salt solution without calcium and magnesium, and heat inactivated fetal bovine serum (FBS) were from Corning (Corning, NY, USA). Charcoal-stripped FBS was from Gemini Bio-Products (West Sacramento, CA, USA). McCoy’s 5A medium modified was from American Type Culture Collection (ATCC, Manassas, VA, USA). l-Glutamine (200 mM) and l-glutamine (200 mM)/penicillin (10,000 U)/streptomycin (10,000 μg) mixtures were from Life Technologies (Grand Island, NY, USA). Dimethyl sulfoxide (DMSO; Hybri-Max™, sterile filtered, BioReagent, suitable for hybridoma, ≥99.7% pure) was from Sigma-Aldrich (St. Louis, MO, USA). Sterile conical tubes, CoolCell cryogenic vial freezing containers, tissue culture (TC)-treated T-flasks, dishes, and 6-well plates, were from Corning. Sterile 1.8 ml cryogenic vials and Mr. Frosty cell freezing containers were from Thermo Fisher Scientific (Pittsburgh, PA, USA).

Tissue culture-treated opaque white 384-well plates, catalog #353988, were from BD Falcon (Bedford, MA, USA), and MicroClime^®^ environmental lids used with these plates were from Labcyte (Sunnyvale, CA, USA). Polypropylene (PP) plates were from Thermo Fisher Scientific Nunc (catalog #264573, non-sterile; and catalog #264574, sterile) and Axygen Scientific (Corning, NY, USA, catalog #P384-240SQ-C). CellTiter-Glo^®^ and Nano-Glo^®^ Dual-Luciferase^®^ Reporter reagent kits were purchased from Promega (Madison, WI, USA). Antineoplastic antibiotic dactinomycin was purchased from AG Scientific (San Diego, CA, USA) for use as a cytotoxic control in cell viability assays. The glucocorticoid dexamethasone was purchased from R&D Systems (Minneapolis, MN, USA). Isopropanol used for pintool and tip washing was certified ACS Plus; ≥99.5% pure grade from Thermo Fisher Scientific.

#### Test Compounds

##### Screening Library

The Spectrum collection of 2,000 biologically active drugs and natural products was purchased from MicroSource Discovery Systems (Gaylordsville, CT, USA) as 10 mM stocks in DMSO in 96-well PP plates. A set of compound plates at 4 mM was generated by plate-to-plate transfer of 10 mM compound stocks into 96-well PP plates and dilution with DMSO. Using a Janus MDT (PerkinElmer, Waltham, MA, USA), the 96-well 4 mM plates were compressed into 384-well format by transfer of sets of four 96-well plates into a single 384-well plate. Each 384-well plate was filled quadrant by quadrant; for example, well A2 of each of four 96-well plates was transferred into wells A3, A4, B3, and B4, respectively, in one 384-well plate. The resulting 384-well compound plates each contained 20 μl of 4 mM compound in DMSO in columns 3–22 (320 compounds/plate), and 20 μl of DMSO in columns 1, 2, 23, and 24 reserved for controls.

All compound plates (and compound powders and solutions in vials) were stored at −30°C in desiccators containing Drierite (W. A. Hammond Drierite Co., Xenia, OH, USA). Prior to storage, plates were sealed with Easy-Peel Heat Sealing Foil covers using an ALPS 50 V Microplate Heat Sealer (Thermo Scientific, Pittsburgh, PA, USA). For compound testing, plates were thawed in desiccators at room temperature (≤12 h) and centrifuged at 250 × *g* for 5 min before removal of the foil cover. Immediately after use, plates were re-sealed, returned to desiccators, and stored at −30°C.

##### PDE Inhibitors

PDE3 and PDE4 inhibitors were purchased from R&D Systems (Minneapolis, MN, USA). Cilostamide, milrinone, (R)-(−)-rolipram, and Ro 20-1724 were part of the Tocris PDE inhibitor set and were obtained as 10 mM stocks in DMSO. Zardaverine, anagrelide (NSC number 724577), CDP 840, trequinsin, piclamilast, YM 976, cilostazol, siguazodan, RS 25344, and ICI 63197 were purchased as powders, from which 50 mM stocks in DMSO were prepared. (Due to limited solubility, cilostazol was dissolved at 40 mM in DMSO.)

##### Zardaverine and Anagrelide Analogs

The following zardaverine analogs were supplied through Ryan Scientific (Mount Pleasant, SC, USA): ChemDiv D216-0257, D216-0505, and D216-0543; Life Chemicals F1967-0306 and F1967-0458; Specs AA-504/34797002 and AG-219/36433016; Vitas-M Laboratory STK359621, STK902092, STK902095, STK931158, STK931862, STK932677, STK943767, STL102657, STL102658, STL141098, STL146832, STL160606, and STL214769. Zardaverine analog imazodan and anagrelide analog quazinone were from Sigma-Aldrich (St. Louis, MO, USA). Compounds were dissolved in DMSO to generate 50 mM stocks except for AA-504/34797002 (16.7 mM), imazodan (12.5 mM), and quazinone (10 mM) due to lack of solubility at higher concentrations.

#### Cell Lines

Except as specified below, all cell lines were grown in DMEM supplemented with 4 mM l-glutamine, 10% FBS, 100 U/ml penicillin, and 100 μg/ml streptomycin at 37°C in an atmosphere of 5% CO_2_. Approval from the Nemours Biosafety Committee and Institutional Review Board was obtained before commencing the cell culture protocols described below (see also Human Subjects Protection).

##### CS242 ERMS (Initial)

Initially, patient-derived CS242 ERMS cells were established in culture in DMEM supplemented with 20% FBS, as described in Robbins et al. (8). Mutation analysis, short tandem repeat (STR) profiling, and fluorescence *in situ* hybridization verified that the established cells had the same genomic characteristics as the original tumor sample, notably homozygous p.G12A mutant *HRAS* and complete loss of maternal chromosome 11 (8). After five passages, cells at 30% confluence were grown for a further 3 days in a T25 flask, followed by expansion into two 10 cm^2^ dishes and continued growth and a media change every 3 days for a total of 9 days, after which time >70% confluence was reached. After removal of media by aspiration, cells were washed with PBS and detached by treatment with 0.25% trypsin. Detached cells were washed in media, pelleted at 200 × *g* for 5 min, and resuspended in freezing medium comprising 90% FBS and 10% DMSO. The resulting master cell bank (at passage 6 following expansion from T25, growth and detachment) was dispensed into 1-ml cryogenic vials at 1–5 million cells/vial, frozen at −80°C for 72 h, and transferred to vapor phase liquid nitrogen storage.

##### CS242 ERMS (Screening)

To generate cells optimized for high-throughput screening (HTS), one vial of frozen cells from the CS242 ERMS (initial) master cell bank was thawed in a 37°C water bath for 2 min, diluted by dropwise addition of 10 volumes of PBS, pelleted at 200 × *g* for 5 min, resuspended in PBS, pelleted, resuspended in medium, and transferred to a T75 flask. Cells were grown to 70% confluence over 27 days, with media changes every 3 days. Cells were then detached by trypsin treatment as described above [see CS242 ERMS (Initial)] and split at 1:20 into T150 flasks, giving 20 individual cultures at passage 7. After growth for a further 27 days with media changes every 3 days, cells reached 70% confluence and were detached and frozen as described above [see CS242 ERMS (Initial)] to give a working cell bank at passage 8.

##### CS242 ERMS (Zardaverine Resistant)

To derive zardaverine-resistant cells, one vial of frozen cells from the CS242 ERMS (screening) cell bank was thawed as described above [see CS242 ERMS (Screening)] and grown to 70% confluence over 3 days in a T75 flask. Cells were detached by trypsin treatment, counted with a hemocytometer using trypan blue exclusion (11), and seeded at 212,000/well in 2 ml of growth media in a 6-well plate. This seeding density was chosen to match 1,250 cells/well in 384-well format, which was found to be optimal for progressive growth during development of the cell viability assay used for screening (Cell Viability Assay Development). After overnight attachment to the plate, the resulting cells at passage 9 were treated with zardaverine at 0.94 μM (~3 × IC_50_ obtained in 384-well plates) and incubated for 3 days, with media (containing zardaverine) changed after 3 days. Cells were detached, split at 1:6 into a new 6-well plate, and the resulting cells at passage 10 were allowed to recover in the absence of zardaverine for 6 days, with a media change after 3 days. Cells were then treated with 15 μM zardaverine for 3 days, detached, split, and expanded into a T75 flask, and allowed to recover for 2 days in the absence of zardaverine. The resulting cells at passage 11 were treated with fresh media containing 15 μM zardaverine and incubated for 4 days at which time cells reached 70% confluence. Cells were detached and frozen as described above [see CS242 ERMS (Initial)] to give a master cell bank at passage 12. One vial of cells from the master cell bank was then used to generate a working cell bank. Cells were thawed into a T75 flask, and immediately maintained in the presence of 15 μM zardaverine in media. Cells were grown for 9 days, with media changes every 3 days, detached, and split at 1:10 into T150 flasks, giving 10 individual cultures at passage 13. After growth for 8 days in the presence of 15 μM zardaverine, with a media (and zardaverine) change after 4 days, cells reached 70% confluence and were detached and frozen to give a working bank of passage 14 zardaverine-resistant CS242 ERMS at 5–10 million cells/ml per vial.

##### Other Cell Lines

Fibroblast cell lines (patient-matched CS242 and normal female) were established as described for CS242 ERMS (initial) culture [see CS242 ERMS (Initial)]. Mutation analysis and STR profiling validated *HRAS* as heterozygous at p.G12A or wild type, respectively (data not shown). Other cancer cell lines not associated with CS were obtained for comparative studies: Rh18 and Rh36 ERMS were a generous gift from Dr. Peter Houghton (Nationwide Children’s Hospital, Columbus, OH, USA); RD ERMS, T24 (bladder carcinoma) and HeLa (cervical carcinoma) were purchased from ATCC (Manassas, VA, USA). Rh36 ERMS and T24 were cultured in RPMI and McCoy’s 5A, respectively, supplemented with 4 mM l-glutamine, 10% FBS, 100 U/ml penicillin, and 100 μg/ml streptomycin.

### Methods

#### Cell Viability Assay Development

##### CS242 ERMS Cell Growth Optimization

Cell growth in 384-well plates was assessed daily for 5 days to determine the optimal starting cell number giving progressive growth suitable for a 72-h cell viability assay. One vial of CS242 ERMS cells was thawed from the screening bank and grown in a T75 flask until confluence was >70%, after which cells were detached using trypsin and counted as described above [see CS242 ERMS (Zardaverine Resistant)].

Cells were twofold serially diluted in media by gentle pipetting in 15-ml conical tubes to give eight concentrations ranging from 250,000 to 1,950 cells/ml. Using an electronic 16-channel pipettor (Finnpipette Novus MCP16), 40 μl of each cell dilution was dispensed into two columns of a TC-treated opaque white 384-well plate, giving a range of 10,000 to 78 cells/well. Columns 1, 2, 23, and 24 were filled with media alone (40 μl/well). Five replicate plates were covered with MicroClime environmental lids (Labcyte) to minimize evaporation and incubated at 37°C, 5% CO_2_ for 24, 48, 72, 96, and 120 h, respectively. A sixth plate was prepared for determination of cell viability at the time of cell seeding. To determine cell viability, plates were allowed to cool to room temperature for 1 h after removal from 37°C incubation, 20 μl/well of CellTiter-Glo was added using a MicroFlo Select microplate reagent dispenser (BioTek, Winooski, VT, USA), and luminescence was measured after an additional 30 min at room temperature using an EnVision microplate reader (PerkinElmer, Waltham, MA, USA). Signal stability was confirmed by re-reading luminescence every 10 min for 2 h.

##### Optimization of Growth of Other Cell Lines

Growth of CS242 fibroblasts, normal female fibroblasts, RD ERMS, Rh18 ERMS, Rh36 ERMS, T24, and HeLa cells was evaluated similarly to that of CS242 ERMS. For each cell line, an optimal starting cell number was selected that gave progressive growth over at least 72 h, and a doubling time comparable to that of CS242 ERMS over this time.

##### Data Analysis

Luminescence values at each time point were converted to cell numbers using a standard curve. A linear standard curve was derived by serial dilution of cells in a 384-well plate (16 twofold dilutions from a top number of 20,000/well) and immediate addition of CellTiter-Glo and measurement of luminescence. Cell numbers were plotted and fit to a non-linear regression exponential growth equation using GraphPad Prism (GraphPad Software, San Diego, CA, USA). This fit enabled assessment of progressive growth and derivation of cellular doubling times. To validate that 72 h of cell growth would be suitable for a cell viability assay, *Z*′-factor analysis (12) was used to evaluate the signal window (defined as the difference between luminescence values corresponding to cell growth and no cells). The *Z*′-factor was calculated according to Eq. 1:

$$Z^{\prime }=1-\frac{\left(3\delta_{\text{c}+}+3\delta_{\text{c}-}\right)}{\left|\mu_{\text{c}+}-\mu_{\text{c}-}\right|},$$

where symbols represent the following: δ_c+_: SD of high controls (with cells); δ_c−_: SD of low controls (without cells); μ_c+_: mean of high controls; μ_c−_: mean of low controls.

#### Compound Screening

##### Assay Quality Control (QC)

Final validation of the CS242 ERMS cell viability assay for HTS was performed by testing QC plates containing the known cytotoxic agent dactinomycin at approximately its IC_50_. The IC_50_ of dactinomycin was determined using cells passaged once from the CS242 ERMS (screening) cell bank [see CS242 ERMS (Screening)]. Cells were thawed, grown to 70% confluence over 3 days in a T75 flask, detached by trypsin treatment, seeded at 1,250 cells in 30 μl of media/well in 384-well opaque white TC plates, and allowed to attach overnight. Dactinomycin was twofold serially diluted from a 160 μM stock to give 16 dilutions in DMSO, which were added to pre-plated cells in two steps: intermediate dilution of 50 nl added by pintool into 20 μl of media, followed by transfer of 10 μl of dactinomycin in media into assay plates to give final concentrations ranging from 100 nM to 3.1 pM. After 72 h of cell growth in the presence of dactinomycin, 20 μl of CellTiter-Glo was added, and cell viability was determined as described above (see Cell Viability Assay Development). Percent viability was determined from luminescence values using high controls (no dactinomycin) and low controls (media alone; no cells), and IC_50_ values were calculated using four-parameter dose–response curve fitting analysis in GraphPad Prism.

Based on a calculated IC_50_ of 0.1 nM, dactinomycin was dispensed into QC plates containing attached cells to give a final concentration of 0.1 nM in all 320 test wells (columns 3–22). DMSO was added to the high control wells (columns 1 and 23) and low control wells (columns 2 and 24; media alone, no cells) by the same two-step addition *via* intermediate dilution as used for dactinomycin. Percent viability was determined as above after 72 h of cell growth.

##### Compound Screening in Cell Viability Assay

The 2,000-compound Microsource Spectrum library was screened using a fresh batch of CS242 ERMS (screening) cells for each HTS run, prepared as described above (see Cell Viability Assay Development). Cells (1,250 in 30 μl media/well) were dispensed into columns 1 and 3–23 of 384-well opaque white TC plates using a MicroFlo Select reagent dispenser equipped with a 5–2,500 μl cassette (BioTek, Winooski, VT, USA). Media alone was added to low control (background) wells in columns 2 and 24. Plates were covered using MicroClime environmental lids (Labcyte), and cells were allowed to attach overnight (≤12 h) at 37°C in an atmosphere of 5% CO_2_.

Compounds were delivered to columns 3–22 of assay plates containing attached cells *via* an intermediate dilution. Using a Janus MDT equipped with a 384-pin slotted pintool (V&P Scientific, San Diego, CA, USA), 50 nl was transferred from storage plates containing 4 mM compound in DMSO (see Screening Library) into 20 μl of media in sterile 384-well PP plates. Ten microliters of media plus compound were added to assay plates by tip transfer using a Janus MDT, giving a final test compound concentration of 2.5 μM. Likewise, DMSO was added to columns 1, 2, 23, and 24 for high and low controls, matching the 0.06% DMSO in the test wells. QC plates containing 0.1 nM dactinomycin (see Assay Quality Control (QC)) were added at the beginning and end of each screening run to monitor plate-to-plate consistency. After 72 h of cell growth in the presence of test compounds, cell viability was determined following addition of 20 μl of CellTiter-Glo (see Cell Viability Assay Development).

Following two replicate screens of the Microsource Spectrum library, percent inhibition of each test compound was averaged, and hits were selected as possessing likely statistically significant activity based on a percent inhibition threshold of >30%. This threshold exceeded 3× the highest percent coefficient of variation of the plate controls observed in the assay plates. Due to the high 5.6% hit rate based on the 30% threshold, selection of compounds for dose–response confirmation was based on a more stringent threshold of >85% inhibition, which reduced the hit rate to 3.1%.

#### Dose–Response Confirmation and Determination of Selectivity

Hits from the Microsource Spectrum library screen in CS242 ERMS were tested in dose–response in CS242 ERMS and CS242 fibroblasts. Using CS242 ERMS and CS242 fibroblast starting cell numbers of 1,250 and 313/well, respectively, cell viability was assayed using the compound screening protocol (see Methods), except that the 320 test wells in each plate comprised serial dilutions of 20 test compounds, one per column. Compounds were cherry picked from 4 mM stocks (see Screening Library) using a Janus Varispan, and twofold serially diluted in DMSO to give 16 dilutions ranging from 4,000 to 0.122 μM, which were added to pre-plated cells in two steps: intermediate dilution of 1.2 μl added by tip into 160 μl of media in PP plates, followed by tip transfer of 10 μl of compound in media into assay plates to give final concentrations ranging from 7.5 μM to 0.23 nM. Dose–response curves and IC_50_ values were determined from percent viability data using four-parameter dose–response curve fitting analysis in GraphPad Prism. Fold selectivity of compound cytotoxicity in CS242 ERMS compared with CS242 fibroblasts was determined by dividing IC_50_ values in CS242 fibroblasts by IC_50_ values in CS242 ERMS.

#### PDE Inhibitor Panel

Phosphodiesterase inhibitors were twofold serially diluted in DMSO from 10, 40, or 50 mM stock solutions (see PDE Inhibitors). Compounds were added to pre-plated CS242 ERMS as described above (see Dose–Response Confirmation and Determination of Selectivity) to give 16 dilutions ranging from a top concentration of 18.8, 75, or 93.8 μM. Cell viability and IC_50_ values were determined according to Section “Dose–Response Confirmation and Determination of Selectivity.”

#### Cell Line Panel

Zardaverine and anagrelide were tested in 16-point dose–response, comprising twofold dilutions from a top concentration of 7.5 μM, in a panel of malignant and non-malignant cell lines (Table 1). Compounds were tested in CS242 ERMS and RD ERMS seeded at 1,250 cells/well, and in CS242 fibroblast, normal female fibroblast, Rh18 ERMS, Rh36 ERMS, T24, and HeLa seeded at 313 cells/well. Cells were exposed to compound for 72 h, and cell viability and IC_50_ values were determined as described above (see Dose–Response Confirmation and Determination of Selectivity). Viability of a separate plate of cells in the absence of test compound was measured at the time of compound addition to determine a luminescence value corresponding to the starting cell number. This allowed for identification of cytostatic effects after 72 h, in which high concentrations of a compound reduce luminescence only as far as a level corresponding to the starting cell number. In contrast, cytotoxic compounds reduce luminescence well below starting values, and ultimately to 0.

**Table 1:** **Cell line panel**.

Cell line	Description	Source	Ras mutation
CS242 embryonal rhabdomyosarcoma (ERMS) (initial)	ERMS; abdominal origin; pediatric	Nemours	Homozygous *HRAS* p.G12A
CS242 ERMS (screening)	ERMS derived from CS242 ERMS primary culture	Nemours	Homozygous *HRAS* p.G12A
CS242 ERMS (zardaverine resistant)	Zardaverine-resistant ERMS derived from CS242 ERMS screening culture	Nemours	Homozygous *HRAS* p.G12A
CS242 fibroblast	Patient-matched skin fibroblast to CS242 ERMS; pediatric	Nemours	Heterozygous *HRAS* p.G12A
RD ERMS	ERMS of muscle origin; pediatric	American Type Culture Collection (ATCC)	Homozygous *NRAS* p.Q61H
Normal female fibroblast	Skin fibroblast; pediatric	Nemours	Wild type *HRAS*
Rh18 ERMS	Non-syndromic ERMS; solid tumor from mouse xenograft; pediatric	Dr. Peter Houghton (Nationwide Children’s Hospital)	Wild type *HRAS*
Rh36 ERMS	ERMS derived from paratesticular relapse; pediatric	Dr. Peter Houghton (Nationwide Children’s Hospital)	Heterozygous *HRAS* p.Q61K
T24	Transitional cell carcinoma of urinary bladder; geriatric	ATCC	Homozygous *HRAS* p.G12V
HeLa	Adenocarcinoma of cervical origin; adult	ATCC	Wild type *HRAS*

#### Mouse Mammary Tumor Virus Promoter (MMTV) Reporter Gene Assay

HeLa cells were seeded in 10 cm^2^ dishes at 2 million cells/dish, in phenol red-free DMEM containing 4 mM l-glutamine and 5% CS-FBS, and allowed to attach overnight. Using Lipofectamine 3000, cells were transfected with one of three combinations of vectors: GL4.36[luc2P/MMTV/Hygro] and pNL1.1.TK[Nluc/TK], pGL4.54[luc2/TK] and pNL1.1.TK[Nluc/TK], or pNL1.1.TK[Nluc/TK] alone. Transfection proceeded for 24 h, after which the cells were harvested and seeded into 384-well opaque white plates by adding 2,500 cells/well in 20 μl fresh media to 5 μl media containing serially diluted zardaverine (15 point, twofold serial dilution, 0.12 nM to 2.0 μM final concentration) and dexamethasone (0 or 100 nM final concentration). After 12 and 24 h, plates were assayed for expression of firefly and NanoLuc luciferases using Nano-Glo^®^ Dual-Luciferase^®^ Reporter, or cell viability using CellTiter-Glo.

### Human Subjects Protection

Ethical approval through the Nemours Institutional Review Board was obtained under protocol Nemours IRB1, IRB #: 2005-051. All patients were enrolled into this approved protocol. Tissue samples were obtained under this protocol as approved. After informed signed consent is obtained from the parent or legal guardian, the individual is assigned an identification number. The patient specific information and the code to the anonymous numerical identifier are kept by the principal investigator and research coordinator in a secure database. All tissue or DNA samples are labeled only with this anonymous identifier. The laboratory identifies samples using this anonymous identifier. Cell lines derived from de-identified samples were subsequently used in the research reported in this manuscript.

## Results

### Cell Viability Assay Development

**Figure 1:**
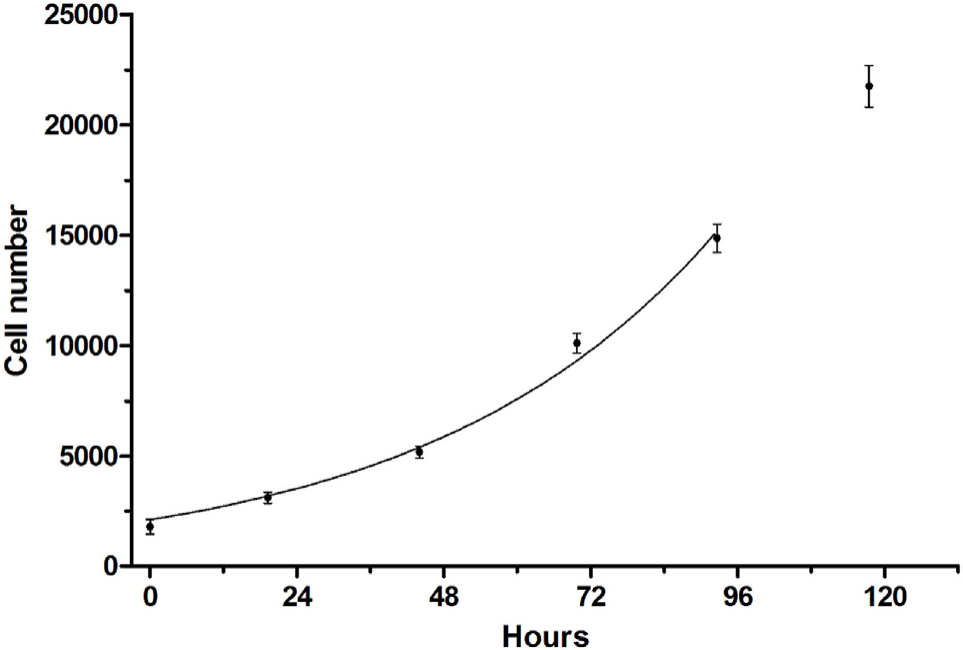
**Growth curve of CS242 embryonal rhabdomyosarcoma cell line optimized for screening**. Cells plated at 1,250/well in 384-well format were assessed for viability using CellTiter-Glo at the times shown. Raw luminescence data were converted to cell number using a standard curve, and data up to 96 h were fit to an exponential growth curve using the exponential growth equation in GraphPad Prism. Data points represent mean ± SD of 48 (0 h) or 32 replicates (remaining time points).

A cell viability assay for CS242 ERMS was developed in which the initial patient-derived CS242 culture was conditioned for HTS. Cell viability was measured using CellTiter-Glo, which gives a luminescent measure of intracellular ATP levels. CS242 ERMS (screening) cells in 384-well microplates gave robust growth over 5 days. The data up to 96 h were fit by non-linear regression to an exponential growth equation in GraphPad Prism and showed an excellent fit to a curve representing exponential growth with a constant doubling time of 32.5 h. The data at 120 h were excluded from the fit because it appeared that cell growth began to slow after 96 h (Figure 1). Similarly, cell viability assays were optimized for CS242 fibroblasts, normal female fibroblasts, RD ERMS, Rh18 ERMS, Rh36 ERMS, T24, and HeLa cells (data not shown). Moreover, *Z*′-factors in excess of 0.75 throughout 5 days of CS242 ERMS growth (Figure S1A in Supplementary Material) were indicative of a consistent and robust assay (12). Cell growth over 4 days in the presence of a range of concentrations of DMSO showed that up to 0.5% DMSO had no effect on cell viability (Figure S1B in Supplementary Material), allowing for a screening protocol to be designed in which test compounds in 100% DMSO may be added to cells *via* a single intermediate dilution in media. Adding CellTiter-Glo to a range of cell dilutions, and reading the resulting luminescence signal at intervals for 2 h thereafter provided a linear cell standard curve, and confirmed signal stability (Figure S1C in Supplementary Material). The cell viability assay was validated for HTS by testing of QC plates containing the cytotoxic agent dactinomycin. Following determination of an IC_50_ of 0.1 nM by dose–response testing of dactinomycin in CS242 ERMS (Figure S2A in Supplementary Material), QC plates were set up by dispensing this concentration of dactinomycin into all 320 test wells of assay plates. Figures S2B,C in Supplementary Material show that percent viability was consistent at approximately 65% within each QC plate and between 12 QC plates tested in separate experiments.

### Compound Screening

**Figure 2:**
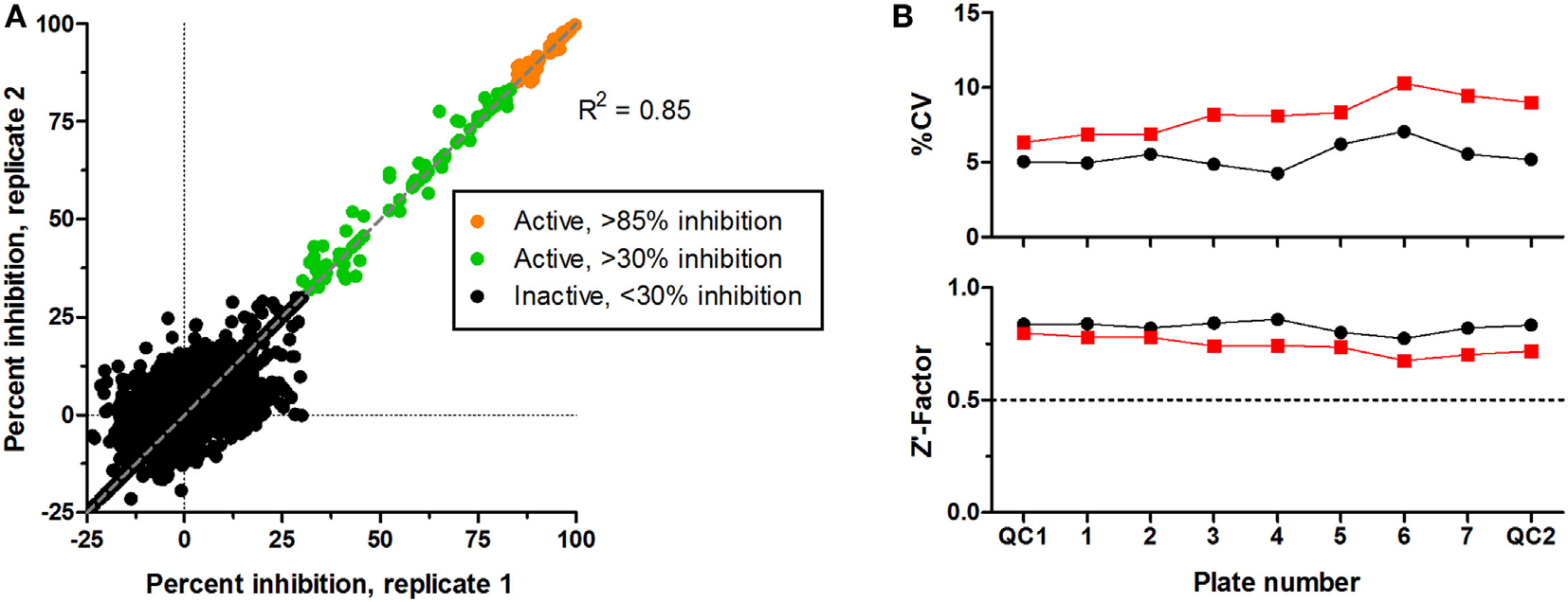
**Screening of Microsource Spectrum library in CS242 embryonal rhabdomyosarcoma**. Compounds were screened at 2,500 nM in duplicate plates. **(A)** Correlation plot on which active compounds are shown based on two inhibition thresholds: >85 and >30% on each plate. Compounds giving <30% inhibition in one or both test wells were considered inactive. Linear regression showed a highly significant correlation between duplicate sets of screening data (*R*^2^ = 0.85). **(B)** Statistical analyses of test plates (numbered 1–7) and quality control (QC) plates (numbered QC1 and QC2) in replicate sets 1 and 2 (black and red, respectively). (Top) %CV, defined as SD ÷ mean expressed as a percentage (calculated from 32 replicate control wells containing cells but no test compound in each plate). (Bottom) *Z*′-factor, calculated as described in Section “Data Analysis.” A threshold *Z*′-factor value of 0.5 is shown by a dotted line on the graph; values >0.5 indicate a robust and reproducible assay.

CS242 ERMS (screening) cells were treated with the Spectrum library of 2,000 FDA-approved drugs, clinical candidates, and pharmacologically active natural products (Microsource Discovery, Gaylordsville, CT, USA). Screening at 2.5 μM of test compound gave an excellent correlation between replicate plates (Figure 2A). A threshold of >85% inhibition of viability (orange symbols) gave 58 hits to retest in dose–response and profile for selectivity in a panel of cell lines. Figure 2B shows that every plate gave highly consistent data statistics. Coefficients of variation (%CV) were all below 10%, well within acceptable limits, and *Z*′-factors in excess of 0.7 confirmed assay robustness (12). Also, not shown in the figure, the control signal was >50-fold above background, and cell number doubling times were consistent at approximately 32 h in all plates.

### Selectivity Profiling of Hits from CS242 ERMS Viability Screen

All 58 hits giving >85% inhibition of CS242 ERMS (HTS) cell viability at 2.5 μM were tested in dose response in CS242 ERMS and patient matched CS242 fibroblasts. Ten compounds showed ≥twofold lower IC_50_ in CS242 ERMS (homozygous p.G12A mutant *HRAS*) than in patient-matched CS242 fibroblasts (heterozygous p.G12A mutant *HRAS*) (Table 2; Figure S3 in Supplementary Material). The PDE inhibitor zardaverine showed remarkable selectivity for CS242 ERMS, inhibiting cell viability with an IC_50_ of 206 nM while displaying no measurable activity in patient-matched non-malignant CS242 fibroblasts. Several topoisomerase inhibitors and cardiotonic agents also showed some selectivity, but follow-up studies were focused on zardaverine by virtue of its striking activity profile.

**Table 2:** **Selective hits from CS242 ERMS screening**.

MicroSource ID	IC_50_ (nM)^^a^^		Fold selectivity^^b^^	Compound name	Pharmacological activity
	CS242 ERMS	CS242 fibroblast			
1506056	206 ± 4.9	>7,500	>36.4	Zardaverine	PDE3 and 4 inhibitor
1505820	1.8 ± 0.1	51.2 ± 13.2	28.4	Topotecan hydrochloride	Antineoplastic; topoisomerase 1
1505708	7.0 ± 1.5	128 ± 17.6	18.3	Epirubicin hydrochloride	Antineoplastic; topoisomerase 2
1504123	12.7 ± 1.5	113 ± 22	8.9	10-Hydroxy-camptothecin	Antineoplastic; topoisomerase 1
1501205	47.7 ± 4.9	196 ± 16.5	4.1	Lanatoside c	Cardiotonic
100749	15.3 ± 1.5	41.5 ± 4.9	2.8	Strophanthidinic acid lactone acetate	Cardiotonic
1500986	111 ± 12.5	280 ± 41.5	2.5	Gitoxin	Cardiotonic
100688	407 ± 5	903 ± 27.7	2.2	Digoxigenin	Cardiotonic
100584	97.8 ± 7.2	196 ± 14.5	2.0	Gitoxigenin diacetate	Cardiotonic
100291	120 ± 4.2	237 ± 18.6	2.0	Strophanthidin	Cardiotonic

*^a^Data represent mean ± SD (*n* = 3)*.

*^b^IC_50_ in CS242 fibroblasts/IC_50_ in CS242 ERMS*.

### Testing of other PDE Inhibitors in CS242 ERMS

**Figure 3:**
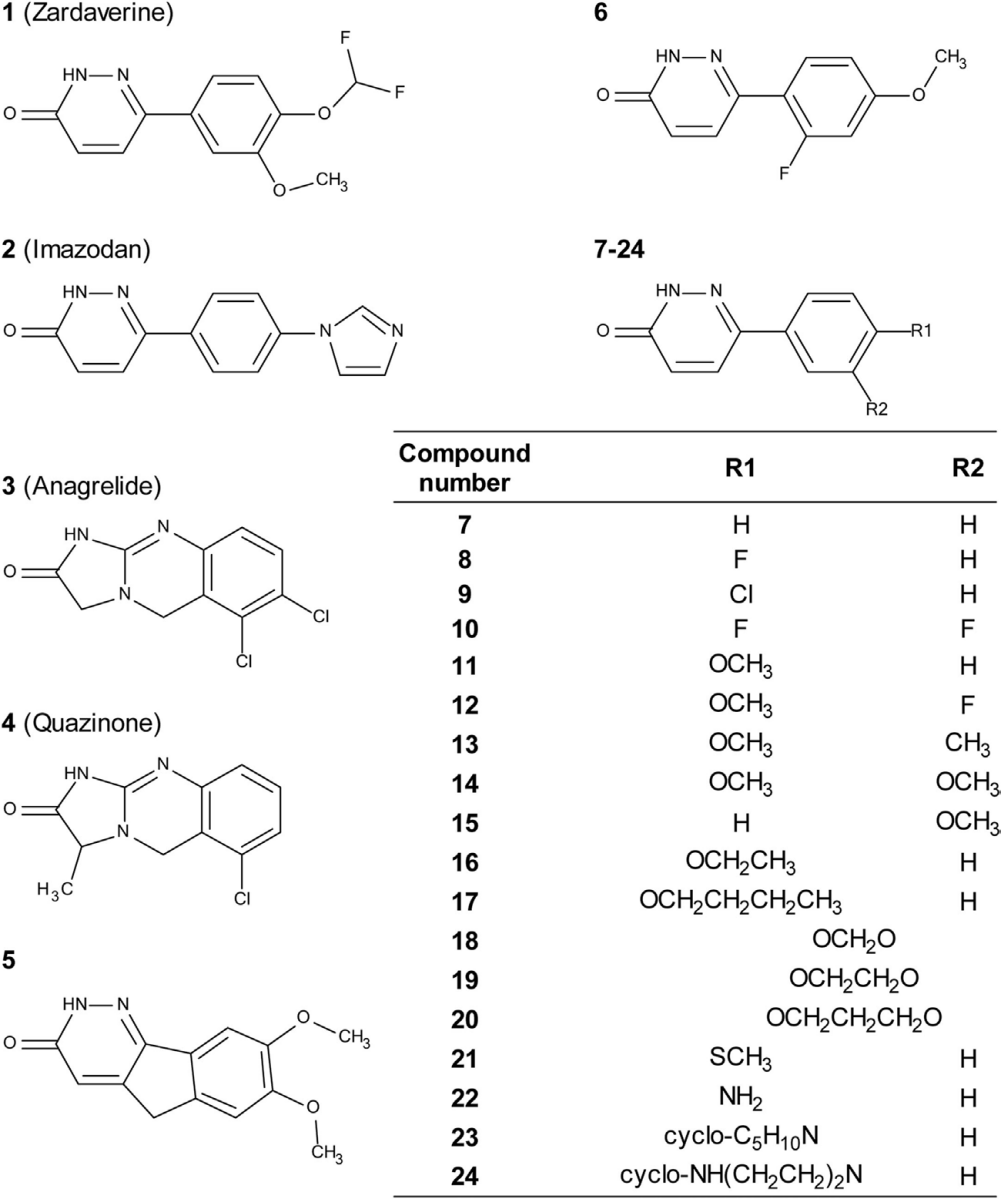
**Structures of zardaverine, anagrelide, and analogs**. Compound numbers correspond with those listed in Table 4.

Zardaverine inhibits PDE3 and 4 (9, 10), so a selection of other PDE3 and PDE4 inhibitors was tested to determine whether they were cytotoxic to CS242 ERMS. Table 3 shows that only zardaverine and anagrelide were potent in CS242 ERMS; a few others had marginal activity, but most showed no measurable inhibition. Taken together, these data suggested that inhibition of the enzymatic activity of PDE3 or 4 may not be sufficient for cell killing in CS242 ERMS and that zardaverine and anagrelide may be also modulating other target(s). The structures of zardaverine and anagrelide shown in Figure 3 share some similar features, suggesting these compounds may interact with a target in CS242 ERMS that is not accessible to the inactive PDE inhibitors.

**Table 3:** **Cytotoxicity of phosphodiesterase (PDE) inhibitors in CS242 embryonal rhabdomyosarcoma (ERMS) compared with reported PDE IC_50_**.

Compound	CS242 ERMS viability	Reported PDE inhibition		
	IC_50_ (nM)^a^	PDE	IC_50_ (nM)	Reference
Anagrelide	68.1 ± 1.6	3	36	(13)
Zardaverine	409 ± 24.3	3/4	1,500/930^b^	(14)
CDP 840	27,800 ± 207	4	12	(15)
Trequinsin	36,500 ± 2,110	3	0.25	(16)
Piclamilast	55,800 ± 1,480	4	1	(17)
YM 976	60,700 ± 3,950	4	2.2	(18)
Cilostamide	>18,800	3	27	(19, 20)
Milrinone	>18,800	3	56	(21)
(R)-(−)-rolipram	>18,800	4	220	(22)
Ro 20-1724	>18,800	4	2,000	(23)
Cilostazol	>93,800	3	200	(24)
Siguazodan	>93,800	3	117	(21)
RS 25344	>93,800	4	0.28	(25)
ICI 63197	>93,800	4	35	(26)

*^a^Data represent mean ± SD (*n* = 3)*.

*^b^Values for PDE3B and PDE4B. Also tested in PDE4D (390 nM)*.

*Anagrelide (13); zardaverine (14); CDP 840 (15); trequinsin (16); piclamilast (17); YM 976 (18); cilostamide (19, 20); milrinone (21); (R)-(−)-rolipram (22); Ro 20-1724 (23); cilostazol (24); siguazodan (21); RS 25344 (25); ICI 63197 (26)*.

### Cellular Selectivity Profiling of Zardaverine and Anagrelide

**Figure 4:**
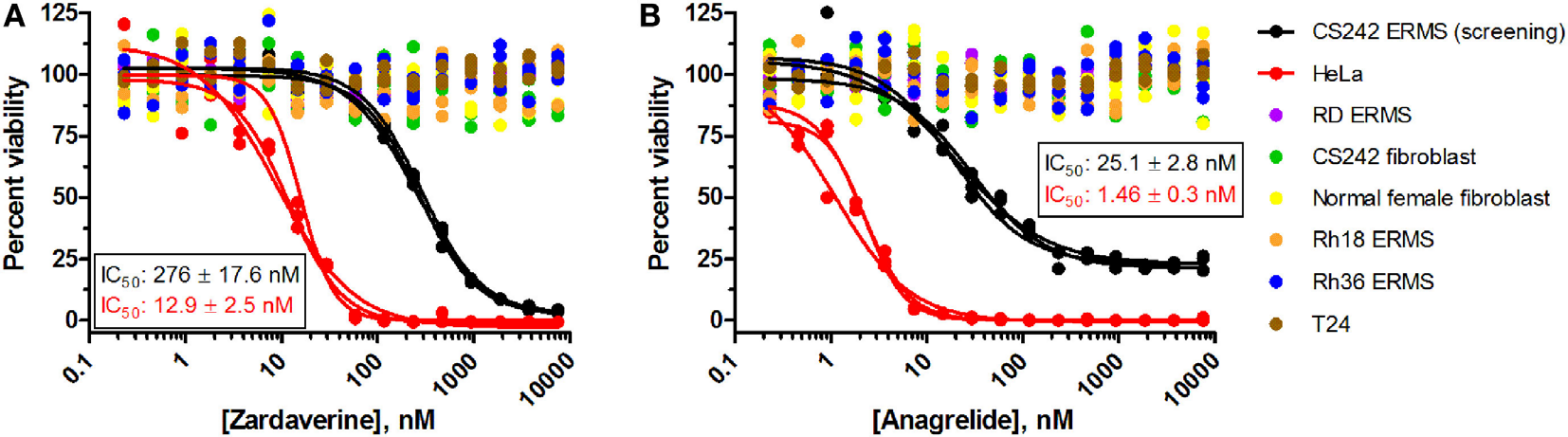
**Profiling of (A) zardaverine and (B) anagrelide in a panel of malignant and non-malignant cell lines**. Table 1 lists the RAS mutation status of each cell line: homozygous mutant, heterozygous mutant, or wild type. Cells were treated with compound for 72 h prior to viability assessment using CellTiter-Glo. Compounds were screened in triplicate; three separate curves are shown, each fit to a single replicate data set. Mean IC_50_ values (±SD) shown on the graphs were obtained by averaging each set of three curve fits.

To further define selectivity for CS242 ERMS, zardaverine and anagrelide were tested in the panel of cell lines listed in Table 1. This panel comprised ERMS harboring various *RAS* mutations or wild type *RAS*; non-malignant fibroblasts harboring heterozygous mutant *HRAS* or wild type *HRAS*; and T24, a bladder carcinoma cell line harboring homozygous mutant *HRAS*. In addition, we included cervical carcinoma-derived HeLa cells, because zardaverine had been reported as cytotoxic in this cell line (27) (Figure 4). The remarkable selectivity of zardaverine for CS242 ERMS over the patient-matched CS fibroblast cell line is shared by anagrelide. Moreover, both compounds showed potent cytotoxicity in HeLa cells, but no discernible effect on any of the other cell lines tested. An interesting difference between the two compounds is that zardaverine showed complete killing of CS242 ERMS at high concentrations, whereas cell viability remained at 25% even at the highest concentrations of anagrelide. The luminescence values determined after 72 h at the highest anagrelide concentrations equal the luminescence measured at the starting cell number on day 0 of the assay. Therefore, the data are consistent with anagrelide exerting a cytostatic rather than a cytotoxic effect in CS242 ERMS, although we have not verified this possibility. The dose–response profile of anagrelide is consistent with the Microsource Spectrum library screening data. Anagrelide was present in the library, but it was not found in the top tier of hits (>85% inhibition, shown in orange in Figure 2A); instead it gave 81% inhibition.

The cytotoxicity profiles of zardaverine and anagrelide among the cell lines tested suggest that *RAS* mutation status is not the primary determinant of compound activity. Activity was observed in both CS242 ERMS and HeLa, yet CS242 ERMS harbors homozygous mutant *HRAS*, whereas HeLa contains wild type *HRAS*. Moreover, neither compound had any effect on the T24 cell line, which harbors homozygous mutant *HRAS*.

### CS242 ERMS Comprises Zardaverine Resistant and Sensitive Subpopulations of Cells

**Figure 5:**
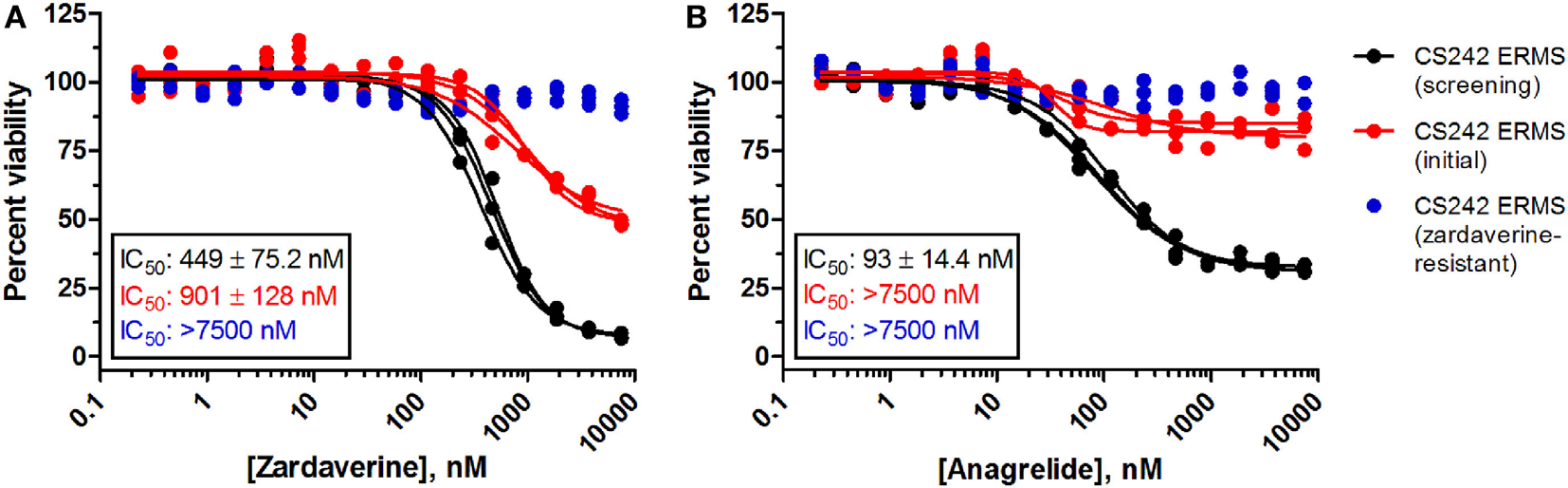
**Zardaverine and anagrelide sensitivity in subpopulations of CS242 embryonal rhabdomyosarcoma (ERMS)**. Graphs show effects of **(A)** zardaverine or **(B)** anagrelide on viability after 72 h exposure to compound of three populations of CS242 ERMS: initial cells from patient-derived tissue (red), cells optimized for screening (black), and screening cells subsequently passaged in the presence of zardaverine (blue). Compounds were screened in triplicate; three separate curves are shown, each fit to a single replicate data set. Mean IC_50_ values (±SD) shown on the graphs were obtained by averaging each set of three curve fits.

Passaging CS242 ERMS in the presence and absence of zardaverine revealed the presence of subpopulations of cells differing in their sensitivity to compound treatment. The CS242 ERMS (screening) population generated by passaging the initial patient-derived culture was the most sensitive. Passaging CS242 ERMS (screening) in the presence of zardaverine generated a CS242 ERMS (zardaverine resistant) population that survived doses of zardaverine lethal to CS242 ERMS (screening). The initial cell population showed intermediate zardaverine sensitivity (Figure 5). Interestingly, cell populations passaged in the presence of zardaverine also acquired resistance to anagrelide, further evidence that the two compounds share a common target. Nevertheless, there may be differences between the mechanism of action; anagrelide differs from zardaverine in that it appears cytostatic in CS242 ERMS (screening) and inactive in CS242 ERMS (initial). Both the rate at which zardaverine resistance appeared during cell culture and the shapes of the dose–response curves in Figure 5 suggest that the CS242 ERMS (initial) cells comprise a mixture of zardaverine-sensitive and zardaverine-resistant populations. The CS242 ERMS (initial) zardaverine dose–response (red curve) appears to level off at 40% cell viability at high concentrations of compound, consistent with killing of only 60% of the cells present after 72 h. Passaging CS242 ERMS (initial) cells to optimize cell growth conditions enriched the zardaverine-sensitive population from 60% of the total population to almost 100% (black curve). Conversely, further passaging of the resultant CS242 ERMS (screening) cells in the presence of zardaverine killed the CS242 ERMS (screening) cells and favored the remaining zardaverine-resistant cell population (blue curve).

Cytogenetic analysis of the subpopulations of CS242 ERMS with varying sensitivity to zardaverine revealed that each population harbored homozygous mutant HRAS and showed the complete loss of maternal chromosome 11 typically found in this CS-derived ERMS, confirming that the sensitive and resistant cell populations originate from the same tumor (data not shown). Nevertheless, the precise nature of the cells within each population and the determinants of zardaverine sensitivity remain to be elucidated.

### Correlation of Activity of Zardaverine and Analogs between CS242 ERMS and HeLa Cells

**Figure 6:**
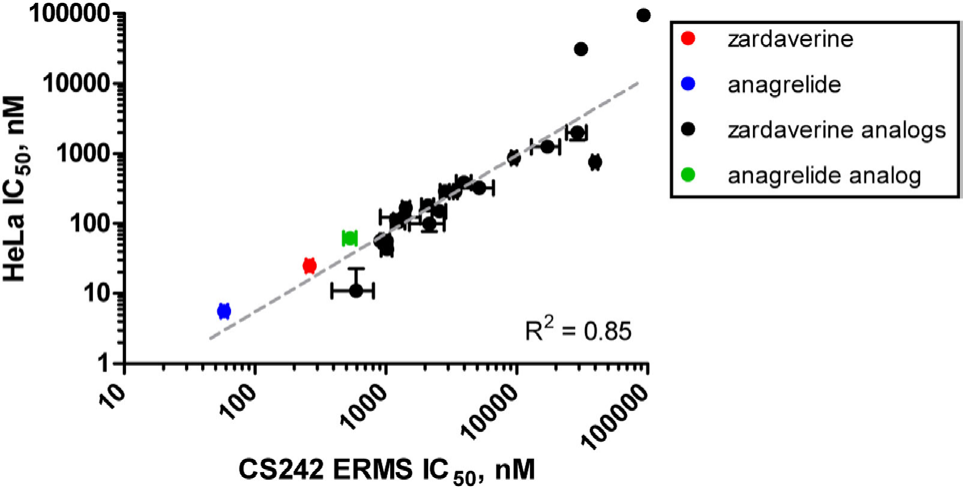
**Correlation of rank-order compound potency between CS242 embryonal rhabdomyosarcoma (ERMS) and HeLa cells**. Anagrelide, zardaverine, and analogs (structures shown in Figure 3) were tested in dose–response for their effects on viability on CS242 ERMS and HeLa cells. Calculated IC_50_ values are listed in Table 4. Each point on the graph represents one compound, plotted according to IC_50_ in CS242 ERMS (*x*-axis) and IC_50_ in HeLa cells (*y*-axis). Data represent mean ± SD for three replicates. (No error bars are shown for the two compounds in the top right-hand corner of the graph because the IC_50_ values were outside the range of compound concentrations tested. In these cases, the *x*–*y* coordinates of the data points shown represent the top concentration tested in each cell line.) Linear regression revealed a highly significant correlation (*R*^2^ = 0.85).

We tested zardaverine in cervical carcinoma-derived HeLa cells because it had been previously reported as cytotoxic in these cells (27). Strikingly, CS242 ERMS and HeLa were the only two cell lines tested in which zardaverine was cytotoxic (Figure 4). The cytotoxicity of zardaverine in HeLa is clearly not mediated through an effect on mutant *HRAS*, given that wild type *HRAS* is present in HeLa cells. This observation raised the possibility that zardaverine cytotoxicity in CS242 ERMS is also not mediated through mutant *HRAS*. Therefore, we decided to explore whether a similar mechanism might account for zardaverine’s cytotoxicity in both CS242 ERMS and HeLa cells. In the absence of specific target information or a measure of target engagement, correlation between structure–activity relationships upon compound testing in two different assays is a reliable indicator that the same target mediates compound activity in both. A substructure search in the PubChem database (28) identified a selection of commercially available zardaverine analogs (Figure 3). These were purchased and tested in both CS242 ERMS and HeLa cells (Table 4). We observed an excellent correlation between IC_50_ values obtained in both (Figure 6), providing evidence that the compounds share a target common to both cell types.

**Table 4:** **Cytotoxicity of zardaverine, anagrelide, and analogs in CS242 embryonal rhabdomyosarcoma (ERMS) and HeLa cells**.

Compound number^a^	CS242 ERMS		HeLa
	IC_50_ (nM)^b^	% Viability (max conc)^c^	IC_50_ (nM)^b^
**3** (anagrelide)	58 ± 4	34.7 ± 1.5	6 ± 1
**1** (zardaverine)	262 ± 15	10.0 ± 0.7	25 ± 3
**4** (quazinone)	535 ± 60	56.4 ± 0.8	62 ± 7
**13**	596 ± 209	12.5 ± 0.0	11 ± 12
**21**	911 ± 13	9.7 ± 0.8	56 ± 6
**17**	1,020 ± 54	11.2 ± 0.3	56 ± 11
**16**	1,020 ± 97	15.3 ± 1.2	43 ± 8
**23**	1,210 ± 62	22.7 ± 0.4	111 ± 6
**12**	1,250 ± 148	49.6 ± 1.8	106 ± 14
**2** (imazodan)	1,370 ± 460	71.5 ± 1.2	124 ± 6
**9**	1,430 ± 99	15.6 ± 1.3	167 ± 9
**20**	2,100 ± 220	31.1 ± 0.8	183 ± 32
**14**	2,160 ± 646	58.6 ± 2.3	99 ± 22
**19**	2,570 ± 308	40.9 ± 1.4	150 ± 17
**6**	2,810 ± 224	53.0 ± 1.3	289 ± 11
**11**	3,420 ± 111	41.2 ± 1.0	289 ± 25
**10**	3,980 ± 515	30.2 ± 0.1	384 ± 52
**18**	5,180 ± 1,480	31.1 ± 19.1	323 ± 29
**15**	9,560 ± 467	53.3 ± 2.7	866 ± 96
**8**	17,200 ± 4,200	50.0 ± 0.0	1,260 ± 58
**7**	29,200 ± 5,040	50.0 ± 0.0	2,010 ± 449
**5**	40,000 ± 2,110	60.0 ± 0.0	756 ± 88
**24**	>31,300	96.8 ± 2.1	>31,300
**22**	>93,800	97.2 ± 1.8	>93,800

*^a^Numbers correspond to structures shown in Figure 3*.

*^b^Data represent mean ± SD (*n* = 3)*.

*^c^Data represent % viability at the maximum concentration of compound tested, which was 93,800 nM except in the case of compounds ***1***, ***3***, and ***4*** (18,800 nM), ***2*** (23,400 nM), and ***24*** (31,300 nM)*.

### Evaluation of Glucocorticoid Receptor (GR) Activation as a Possible Target of Zardaverine

A search of the PubChem database suggested that inhibition of GR activation might mediate cell killing by zardaverine in HeLa cells. Aside from PDE inhibition, the only sub-micromolar activity of zardaverine reported as confirmed in PubChem is decrease in HeLa cell number (29). Inhibition of GR activation in engineered HeLa cells was reported as inconclusive (30), but close inspection of the dose–response data suggested to us that zardaverine was active in this screen, with an IC_50_ of 0.3 μM closely matching our data. Interestingly, the PDE inhibitors anagrelide and cilostamide were also tested in the GR activation screen and were highly active and inactive, respectively, consistent with our CS242 ERMS results. We were encouraged that HeLa cells in the GR activation screen were treated with test compounds for only 18 h, because we had observed that the cytotoxic effects of zardaverine in HeLa cells were not apparent before 24 h. Therefore, we reasoned that zardaverine may inhibit GR activation as a precursor to induction of cell death, and it might be possible to observe an effect of zardaverine on GR activation prior to cell killing.

We selected the inducible GL4.36[luc2P/MMTV/Hygro] vector to determine whether zardaverine inhibited gene expression driven by GR activation. This vector expresses firefly luciferase under the control of the MMTV, which contains steroid response elements that activate gene expression in response to glucocorticoids such as dexamethasone. A protein degradation sequence (PEST) (31) tagged onto the luciferase results in protein turnover with a half-life of approximately 1 h (32), thereby ensuring a rapid assay response. The pGL4.54[luc2/TK] vector served as a control for global effects on gene expression. This vector expresses firefly luciferase under the control of a herpes simplex virus thymidine kinase (HSV-tk) promoter, which does not require induction by dexamethasone. Luciferase expressed by pGL4.54[luc2/TK] lacks a PEST sequence, resulting in a 4-h half-life (32). Both firefly luciferase expressing cell groups also contained the pNL1.1.TK[Nluc/TK] vector expressing NanoLuc luciferase from the HSV-tk promoter, and in addition we included a set of control cells expressing only NanoLuc and not firefly luciferase. We initially thought to utilize NanoLuc as an internal control to monitor consistency between cell preparations, but its extreme stability and long half-life of approximately 7 days make it unsuitable (33).

**Figure 7:**
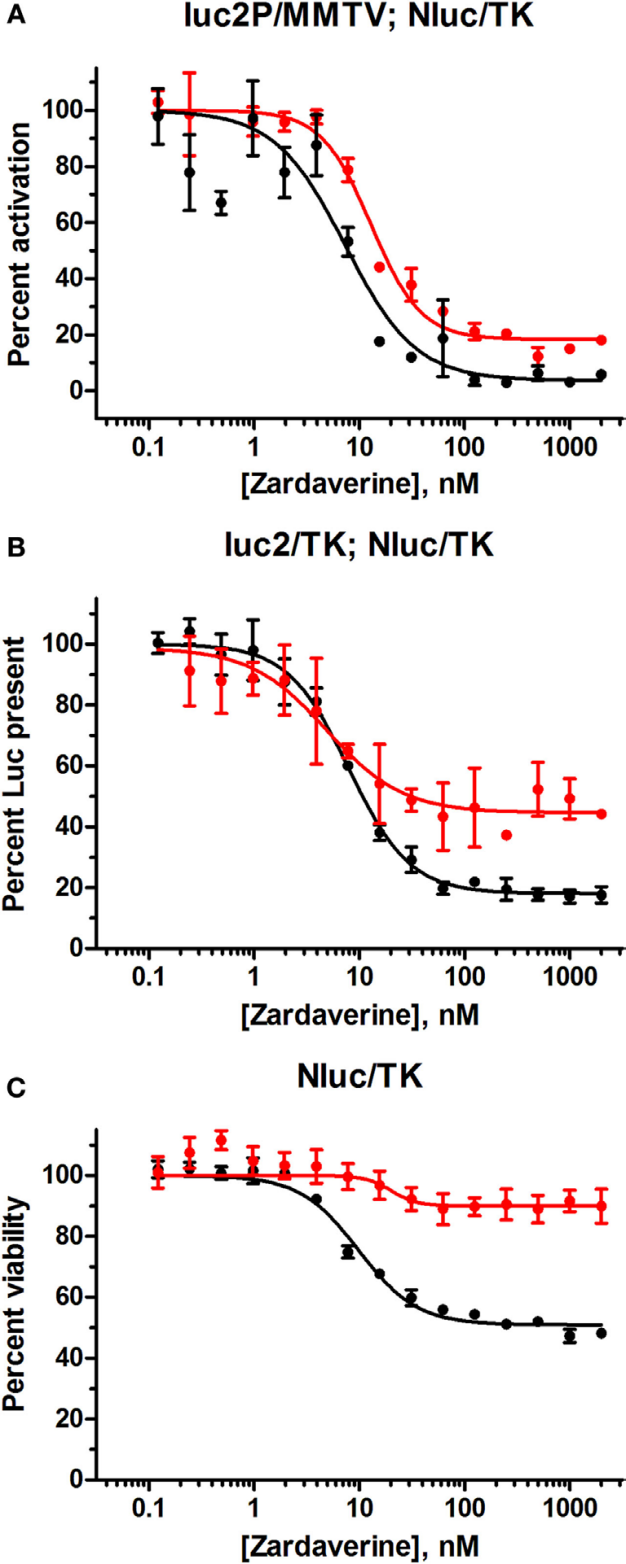
**Zardaverine dose–response testing in HeLa reporter assays**. **(A)** HeLa cells transfected with GL4.36[luc2P/MMTV/Hygro] and pNL1.1.TK[Nluc/TK] were assayed for firefly luciferase activity 12 h (red) and 24 h (black) after treatment with zardaverine in the presence of 100 nM dexamethasone; **(B)** HeLa cells transfected with pGL4.54[luc2/TK] and pNL1.1.TK[Nluc/TK] were assayed for firefly luciferase activity with OneGlo 12 h (red) and 24 h (black) after treatment with zardaverine; **(C)** HeLa cells transfected with pNL1.1.TK[Nluc/TK] were assayed for cell viability with CellTiter-Glo 12 h (red) and 24 h (black) after treatment with zardaverine. Data are represented as percent activity compared to controls without zardaverine (*n* = 3, average ± SEM; in some cases the error bars are obscured beneath the plot symbol).

HeLa cells transfected with the GL4.36[luc2P/MMTV/Hygro] vector gave minimal luciferase expression in the absence of dexamethasone. Titration of dexamethasone gave a concentration-dependent increase in luciferase expression up to a maximal level at 100 nM and above (Figure S4 in Supplementary Material). As expected, cells containing pGL4.54[luc2/TK] gave robust luciferase expression in the absence of dexamethasone. Therefore, the effect of zardaverine on luciferase expression in response to GR activation was determined in cells transfected with GL4.36[luc2P/MMTV/Hygro] and treated with 100 nM dexamethasone. Zardaverine showed a strong effect after only 12 h, and after 24 h potently and completely reversed dexamethasone activation of luciferase expression with an IC_50_ of 7.7 nM (Figure 7A). However, substantial reduction of expression in cells under the control of pGL4.54[luc2/TK] was also observed after 12 h (Figure 7B, red), and by 24 h this constitutive luciferase expression was almost completely inhibited with an IC_50_ of 8.0 nM (Figure 7B, black). Zardaverine treatment of control HeLa cells containing only the NanoLuc vector showed the expected minimal effect on cell viability after 12 h, and a partial effect after 24 h (Figure 7C). Levels of the stable NanoLuc luciferase were unaffected by zardaverine in all three cell populations (representative data shown in Figure S5 in Supplementary Material). Based on the minimal difference between the effects of zardaverine on dexamethasone-dependent and dexamethasone-independent luciferase expression, we concluded that zardaverine does not specifically inhibit GR activation, but instead exerts a global effect on gene expression prior to cell killing. The longer time required to completely inhibit constitutive as compared with inducible luciferase expression [24-h curve in Figure 7B (black) resembles 12-h curve in Figure 7A] is most likely related to the longer half-life of the constitutively expressed luciferase.

## Discussion

The purpose of screening CS-derived ERMS cells harboring mutant HRAS using a simple phenotypic readout of cell viability was to identify compounds adversely affecting cell viability by unanticipated mechanisms of action, and thereby uncover novel targets to advance understanding of ERMS biology and improve treatment options. Our discovery of potent cell killing by zardaverine and apparent cell growth arrest by anagrelide offers the possibility of repurposing these compounds for treatment of ERMS in children with CS. Both compounds were also cytotoxic in HeLa cells but had no effect on any of the other cell lines tested. These compounds were originally developed as PDE inhibitors (9, 10): zardaverine underwent clinical testing as an asthma therapeutic, while anagrelide was approved for treatment of essential thrombocytosis (34, 35). However, the cell growth arrest and killing we observed in CS-derived ERMS may be mediated through another mechanism in addition to inhibition of the enzymatic activity of PDEs. We found that many PDE inhibitors equally potent as enzyme inhibitors compared to zardaverine had no effect on CS242 ERMS (Table 3). However, these results should be interpreted with caution. We have not confirmed that the PDE inhibitors we purchased are active in our hands against the isolated enzymes. Moreover, the PDEs are a large family of enzymes and subtypes, with wide-ranging biological functions that depend on cellular localization as well as structural features. PDEs and their properties have been described extensively in several excellent reviews (36–38). A comprehensive study of zardaverine in a panel of PDEs found that zardaverine was reasonably but not absolutely selective for PDE3 and PDE4. The most potent inhibition was against PDE3B, PDE4B, and PDE4D, with IC_50_s of 1500, 930, and 390 nM, respectively, but modest inhibition (IC_50_s of 14–160 μM) was observed against PDEs 5A, 8A, 10A, and 11A (14). Therefore, based on the information in Table 3 alone, we cannot rule out that the cytotoxicity of zardaverine in CS242 ERMS is mediated through inhibition of a PDE3 or PDE4 subtype in a specific cellular location, or another PDE not inhibited by the other compounds tested. Nevertheless, these results raised the intriguing possibility that zardaverine and anagrelide act on a hitherto unexpected molecular target, either through a mechanism in which PDE inhibition is not involved at all, or mediated by a cooperative effect between PDE inhibition and another target present in CS242 ERMS and HeLa but not the other cell lines tested.

A recent report published while this manuscript was in preparation provides compelling evidence for modulation of a second target mediated through PDE inhibition, at least in the squamous cell carcinoma-derived HeLa cell line (39). Zardaverine, anagrelide, and a zardaverine analog dubbed DNMDP were cytotoxic to HeLa and certain lung cancer and melanoma cell lines but had no effect on most of a panel of 766 cancer cell lines. Consistent with our results, other equally potent inhibitors of PDE enzymatic activity were not cytotoxic. Chemogenomic analysis revealed a strong correlation with expression of both *PDE3A* and *SLFN12* in the sensitive cell lines. The overall conclusion of the study was that DNMDP exerts two functional effects upon binding to the enzyme active site of PDE3A: in addition to the “expected” inhibition of the enzymatic PDE activity, this compound enhances binding of SLFN12 to PDE3A through an allosteric effect. How PDE3A recruitment of SLFN12 results in tumor cell death is still to be determined.

Although we do not yet have direct evidence for a connection between PDE3A–SLFN12 binding and zardaverine cytotoxicity in CS242 ERMS, our findings are consistent with this interaction being important in CS242 ERMS as well as HeLa. Compound testing results suggest that zardaverine, anagrelide, and analogs cause cytotoxicity and cell growth arrest in CS242 ERMS and HeLa through the same mechanism. We determined potency of a series of analogs in both cell types; correlation of the rank-order potency of a series of compound analogs between two assays is generally a robust and reliable indicator of a common mechanism of action. We purchased a substantial number of analogs closely related structurally to zardaverine and one analog of anagrelide (Figure 3). The effects of zardaverine, anagrelide, and these analogs on cell viability correlated strongly between CS242 ERMS and HeLa (Figure 6), suggesting that all compounds act on the same target(s) in both cell lines.

An earlier study also reported cytotoxicity of zardaverine in HeLa cells but did not suggest the involvement of a non-PDE target (27). The authors attributed cell killing to PDE inhibition alone. However, these conclusions were based on activity of six PDE inhibitors among hits from the LOPAC library (Sigma, St. Louis, MO, USA). The LOPAC library contains another 25 known PDE inhibitors, and there is no mention of cytotoxicity in HeLa cells associated with these compounds.

In a third study of zardaverine in cancer, treatment of hepatocellular carcinoma cell lines led to potent and selective cytotoxicity (40). Consistent with our results, zardaverine killed certain cell lines but not others, whereas other PDE inhibitors had no cytotoxic effect. Selective killing by zardaverine in hepatocellular carcinoma cell lines was linked to cell cycle arrest and subsequent induction of apoptosis by a mechanism independent of inhibition of PDE enzymatic activity. It was postulated that cell cycle arrest at G0/G1 and subsequent apoptosis was due to low expression of retinoblastoma protein (Rb) and perturbation of Rb-regulated signaling. To determine whether Rb mediates zardaverine cytotoxicity in CS242 ERMS, we measured levels of total and phosphorylated Rb in zardaverine-sensitive CS242 ERMS as well as in zardaverine-resistant CS242 ERMS and patient-matched fibroblasts. However, using western blotting, we saw no significant differences between compound treated and untreated cells (data not shown).

To reach beyond the peer-reviewed literature, we searched the PubChem database (http://pubchem.ncbi.nlm.nih.gov) for further insights into the mechanism of cytotoxicity of zardaverine and anagrelide in CS242 ERMS and HeLa. Interestingly, zardaverine showed inhibitory activity in a HeLa cell-based reporter gene screen for GR activation, suggesting that GR signaling might be involved in cell killing by zardaverine (30). To test this hypothesis, we set up a reporter gene assay in which luciferase expression in HeLa cells was dependent on GR activation, and a counterscreen comprising constitutive expression of luciferase. We found that zardaverine inhibited inducible and constitutive luciferase expression with almost identical IC_50_ values and dose–response profiles (Figure 7), leading us to conclude that the effects on gene expression preceding cell killing by zardaverine are non-specific and not mediated through a specific effect on GR activation.

Zardaverine is a potent cytotoxic agent in CS242 ERMS and appears to be quite selective in its action. No other sarcoma cell lines were affected by zardaverine, neither in our panel (Figure 4), nor in the panel of 766 cell lies tested by de Waal et al. (39). However, what is not clear is whether the susceptibility of CS242 ERMS is shared by ERMS arising from other CS patients. The Nemours CS registry and tissue repository contains ERMS from eight patients (41), but to date the only established culture is that from patient CS242. Moreover, we have shown that CS242 contains a subpopulation of cells that are not affected by zardaverine (Figure 5). Further study of the mechanism of action of zardaverine in CS242 ERMS should provide insights into the biology of CS-derived ERMS, but the cellular susceptibilities revealed will not be exploitable therapeutically until we establish the frequency with which they arise in other cases of CS-derived ERMS. If we are able to extend the study of zardaverine to additional patient samples, the selectivity of zardaverine in CS242 ERMS may offer the possibility of a therapeutic option tailored to children suffering from CS-derived high-risk ERMS.

## Author Contributions

DC, KR, and KD designed and performed experiments, analyzed and interpreted data, and assisted with manuscript writing; RS performed experiments and analyzed data; DS performed experiments; KG, EK, KS-C, and AN formulated strategy and directed research; AN wrote the manuscript; all authors approved the final version of the paper.

## Conflict of Interest Statement

The authors declare that the research was conducted in the absence of any commercial or financial relationships that could be construed as a potential conflict of interest.
