# Supplementary material for: Cytotoxicity of Zardaverine in Embryonal Rhabdomyosarcoma from a Costello Syndrome Patient
Source: Front Oncol. 2017 Apr 3;7:42. doi: 10.3389/fonc.2017.00042 (PMC5376947; doi:10.3389/fonc.2017.00042)
Supplement: Supplementary file 1 [file Data_Sheet_1.PDF]

frontiers

Supplementary Material

Selective cytotoxicity of zardaverine in embryonal rhabdomyosarcoma from a Costello syndrome patient

Donna M. Cartledge,[1] Katherine M. Robbins,[2,3] Katherine M. Drake,[1] Rachel Sternberg,[1,4] Deborah L. Stabley,[2] Karen W. Gripp,[5] E. Anders Kolb,[6] Katia Sol-Church,[2] Andrew D. Napper.[1*]

*** Correspondence:** Corresponding Author: Andrew D. Napper, Nemours Center for Childhood Cancer Research, Nemours/A.I. duPont Hospital for Children, 1701 Rockland Road, Wilmington, DE 19803, USA

napper@nemoursresearch.org

Supplementary Figures

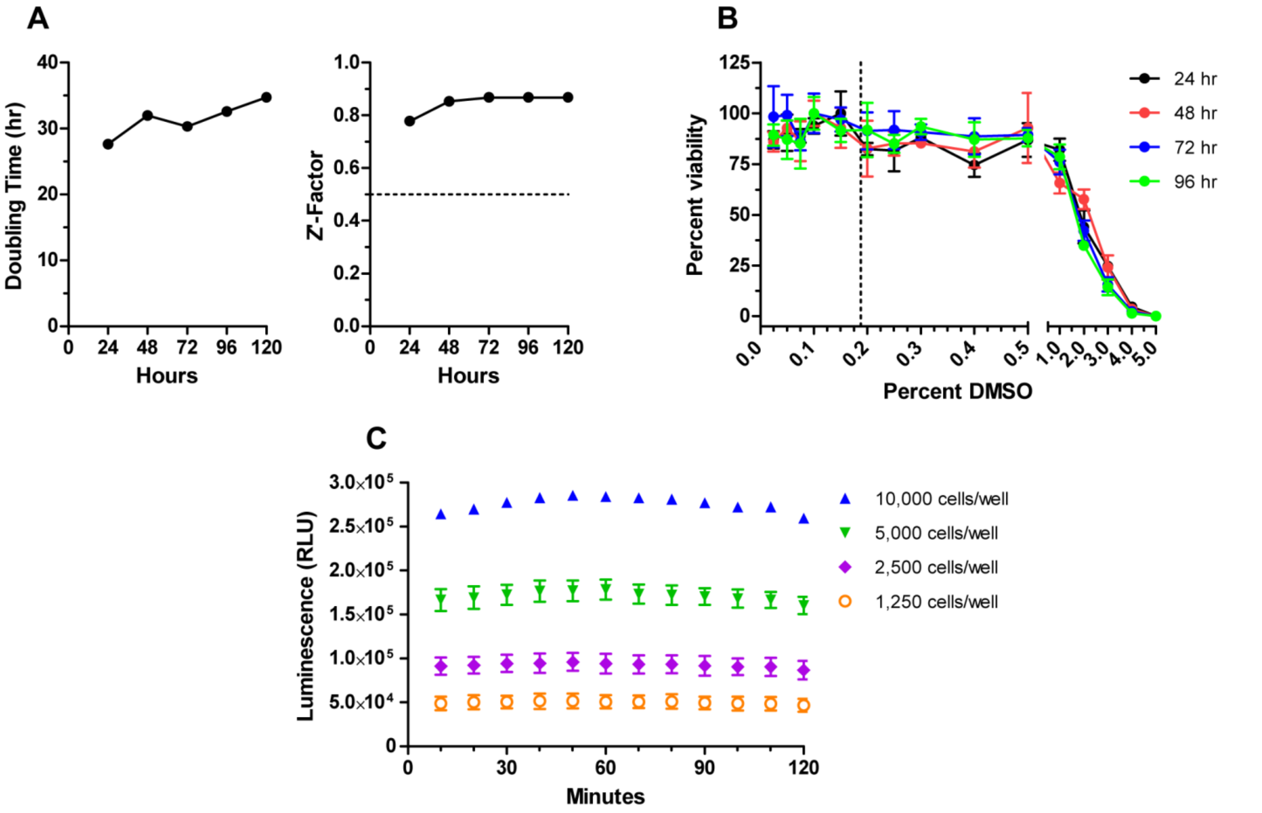

Supplementary Figure 1. Optimization of CS242 ERMS cell viability assay: determination of (A) doubling time and Z'-factor, (B) DMSO tolerance, and (C) signal stability. (**A-C**) Cells were plated at 1250/well in 384-well plates and viability at specified times thereafter was assessed using CellTiter-Glo. (**A**) Cellular doubling times and Z'-factor values at time intervals up to 120 hr. Luminescence values were derived from 32 replicates at each time point, and cellular doubling times and Z'-factors were calculated as described in Section 2.2.1.2. (**B**) Viability of cells treated with 0-5% DMSO was determined after 24-96 hr of exposure (n=3; mean ± SD). Vertical dashed line indicates DMSO concentration of 0.19% used in the HTS assay format. (**C**) Luminescence was measured every 10 min for 2 hr immediately after plating cells at concentrations shown (n=3; mean ± SD).

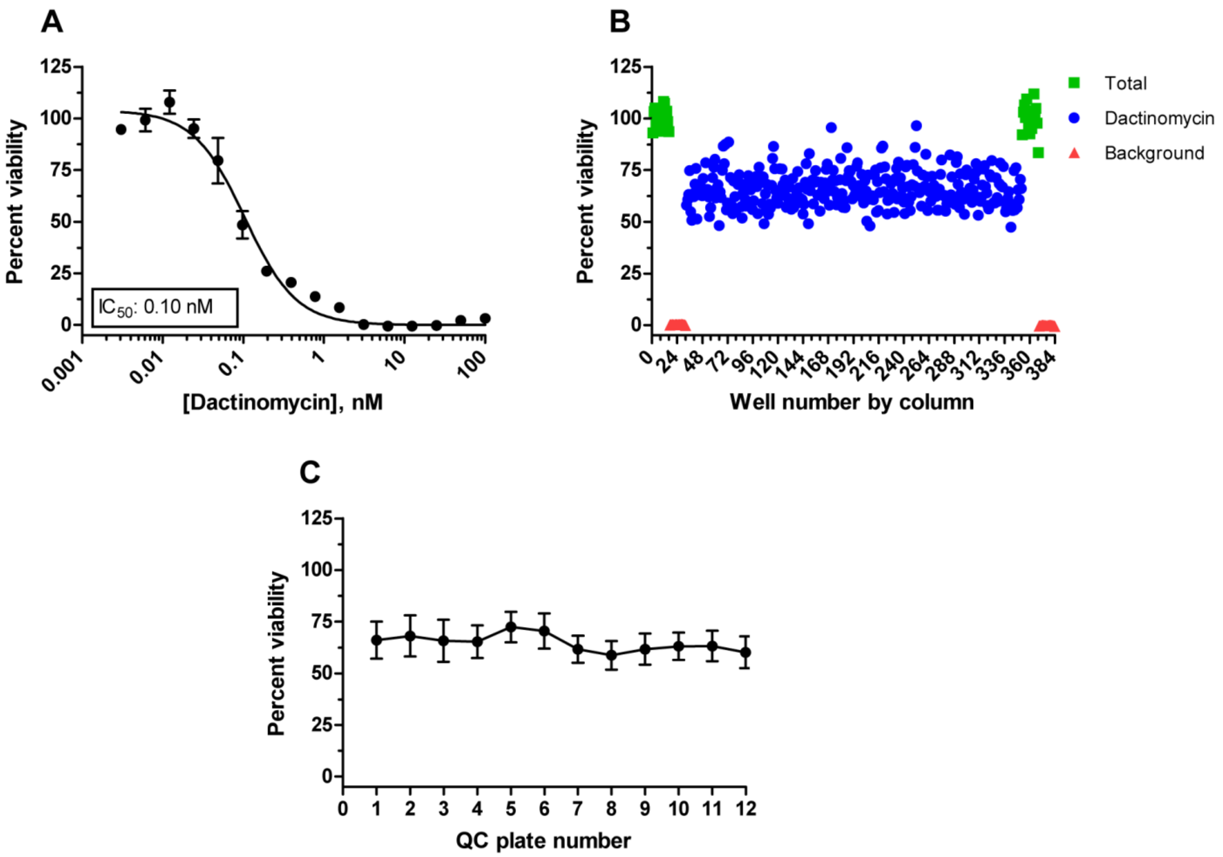

Supplementary Figure 2. Assay quality control (QC). (**A**) Positive control compound dactinomycin IC_{50} determination after treatment of CS242 ERMS (1250 cells per well) for 72 hr. Percent viability was calculated from CellTiter-Glo luminescence measurements (n=3; mean ± SD). (**B**) Representative QC plate. Dactinomycin at 0.1 nM was added to all 320 test wells (blue). (**C**) Mean dactinomycin percent inhibition averaged within each of 12 QC plates (mean ± SD; n=320 per plate).

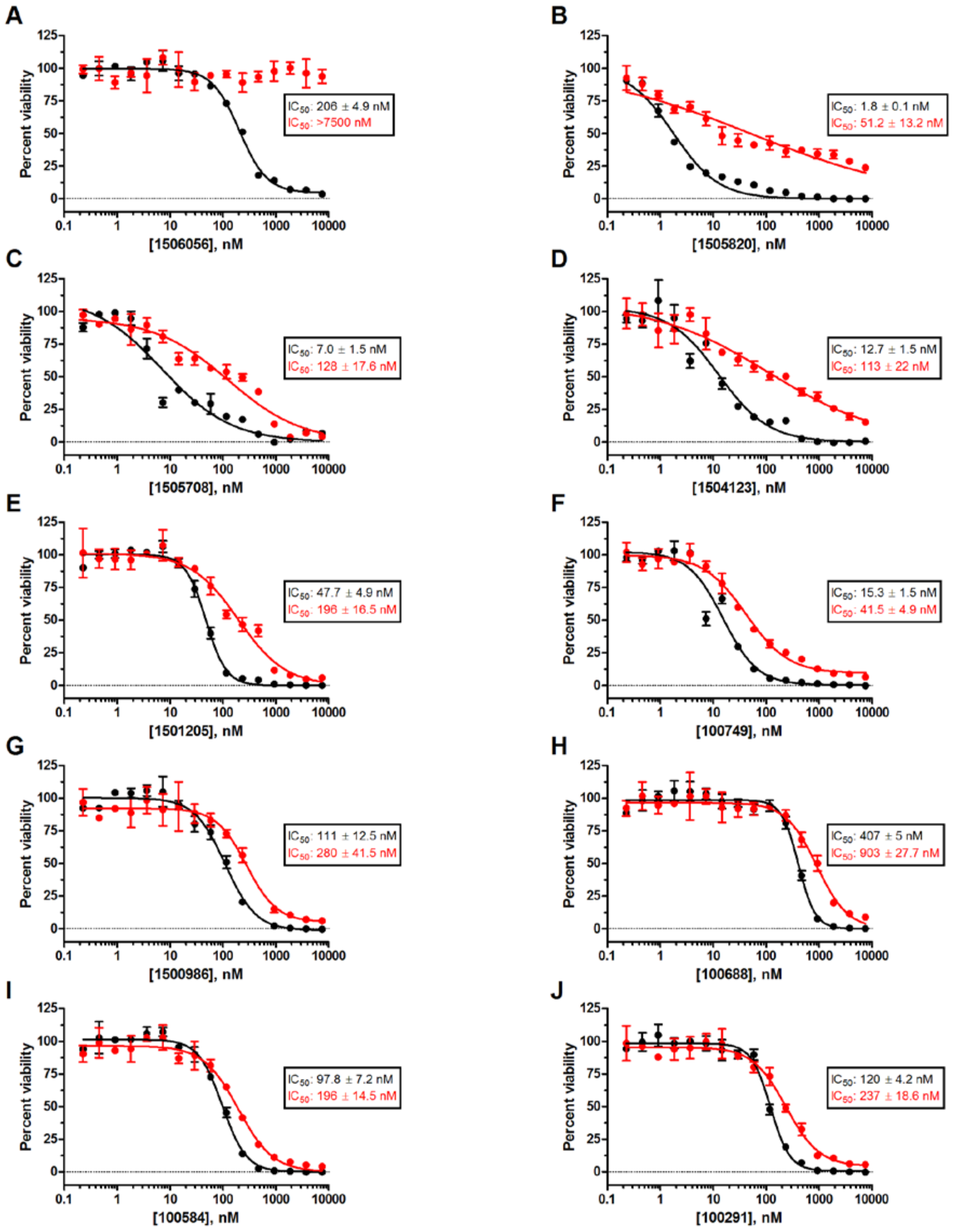

Supplementary Figure 3. Profiling of selective hits from pilot screening in CS242 ERMS (black) and patient-matched CS242 fibroblast (red) cell lines. Cells were exposed to compound for 72 hr prior to measurement of viability using CellTiter-Glo. Extent of cell killing was determined by dose-response with mean IC_{50} values calculated from percent viability of triplicate samples. Data represent mean ± standard deviation. Table 2 lists the IC_{50} values for each compound in each cell line.

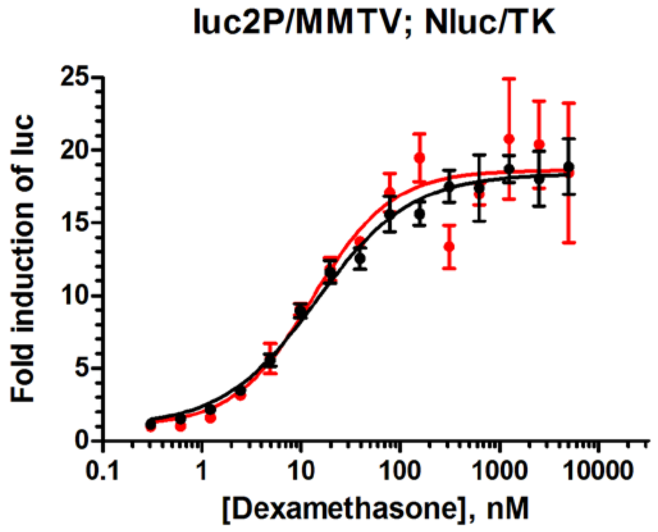

Supplementary Figure 4. Induction of luciferase by dexamethasone in HeLa cells transfected with the pGL4.36[luc2P/MMTV/Hygro] vector. HeLa cells transfected with GL4.36[luc2P/MMTV/Hygro] and pNL1.1.TK[Nluc/TK] were assayed for firefly luciferase activity 12 hr (red) and 24 hr (black) after treatment with dexamethasone. Data are represented as fold induction of luciferase activity compared to controls without dexamethasone (n = 3, average ± SEM).

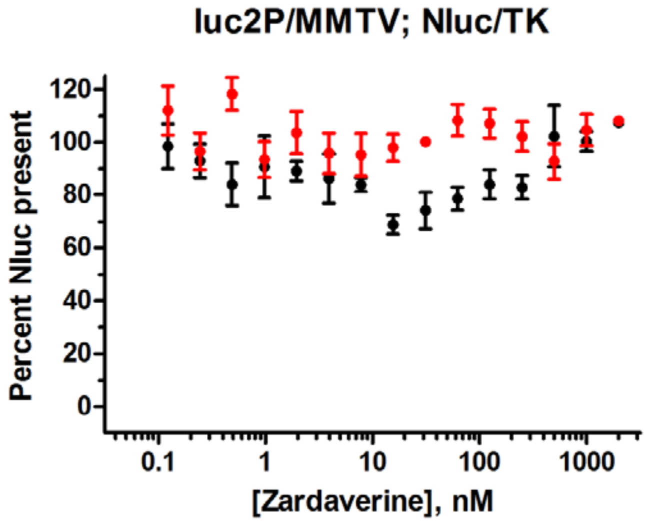

Supplementary Figure 5. Zardaverine dose-response testing for NanoLuc luciferase activity in HeLa reporter assays. HeLa cells transfected with GL4.36[luc2P/MMTV/Hygro] and pNL1.1.TK[Nluc/TK] were assayed for NanoLuc luciferase activity 12 hr (red) and 24 hr (black) after treatment with zardaverine in the presence of 100 nM dexamethasone. Data are represented as percent NanoLuc luciferase activity compared to controls without zardaverine (n = 3, average ± SEM).
